# Guest Editorial: Biomarkers of Perfluorinated Chemicals and Birth Weight

**DOI:** 10.1289/ehp.10923

**Published:** 2007-11

**Authors:** David A. Savitz

**Affiliations:** Department of Community and Preventive Medicine, Mount Sinai School of Medicine, New York, NY 10026, E-mail: david.savitz@mssm.edu

Two articles (Apelberg 2007; Fei 2007) appearing in this issue of *Environmental Health Perspectives* evaluate the relationship of perfluorooctanoate (PFOA) and perfluororooctane sulfonate (PFOS) with birth weight; they constitute the first rigorous epidemiologic studies to address this topic. The ubiquity of these chemicals in the environment and in humans and the growing concerns about potential health effects from such toxicants make these studies a valuable addition to the health effects literature. The ultimate implications of these studies concerning etiologic relationships with human health remain to be elucidated, but they raise two important issues: the interpretation of small shifts in birth weight, and the potential for shared determinants of exposure biomarkers and biological indicators of health outcome.

Birth weight offers many advantages as an end point for studies of environmental agents. It is measured easily and accurately, shows substantial variability, and has been proven to be sensitive to environmental insults, most notably tobacco smoke (Wilcox and Russell 1983; Windham et al. 2000). Because birth weight is measured on a continuous scale, even moderate-size studies often have excellent statistical power to detect small decrements. On the other hand, such shifts in birth weight, even if ultimately proven to be causal, reflect variation within the normal range of the distribution, in which there appears to be few or no direct consequences for infant mortality or morbidity (Wilcox 2001). Even when there are relatively large shifts in birth weight, as associated with altitude or cigarette smoking, there do not appear to be adverse effects mediated by the shift in mean birth weight per se. Although shifts in the entire distribution would be expected to increase the number of births at the extreme low end, in fact it seems that influences on the dominant part of the birth weight distribution operate separately from determinants of the extreme residual end of the scale (Wilcox 2001; Wilcox and Russell 1983). When studies measure changes in mean birth weight, the results reflect only the dominant distribution—the normal ranges. The same is true for gestational age: Changes in mean gestational age at birth tell us little about changes in the risk of preterm birth, and instead reflect changes in the normal 38- to 40-week range where most births occur. The number of births falling into the residual tail of the distribution determines the clinical and public health outcomes of importance. In contrast, blood pressure, body mass index (BMI), and blood glucose levels appear to affect risk of cardiovascular disease on a continuous scale across the full range, so that shifts in the entire distribution are highly influential (Rose 1985).

In the study by Apelberg et al. (2007), the strongest association was found between PFOA and birth weight, with a log unit change (2.7-fold increase) in exposure associated with a 104-g [95% confidence interval (CI), −5 to 213 g] reduction in weight. A log unit change in PFOS predicted a 69-g (95% CI, −10 to 149 g) reduction. In the study by Fei et al. (2007), PFOA was associated with birth weight, with a decrement of 10.6 g (95% CI, 0.5 to 20.8) per nanogram per milliliter change in exposure, and essentially no association was found for PFOS. Neither study provided support for a change in risk of low birth weight as conventionally defined (< 2,500 g), presumably reflecting modest shifts in the overall distribution without any enhanced effect on the tail. Given the differences in which agent was implicated, variation in ranges of exposure studied, and modest effect sizes, the evidence linking PFOA or PFOS and birth weight remains ambiguous. But if the link is confirmed, it would suggest biological (not necessarily pathological) effects of exposure in humans. The reported variations in head circumference and ponderal index share the same concerns—uncertain implications of small variation in the normal range (Apelberg et al. 2007).

A second concern when relating a biomarker of exposure to a measure of normal biological variation is the possibility that both reflect shared maternal physiology. In the case of exposure to PFOA and PFOS in communities lacking distinctive environmental sources, as in these two studies, interindividual variation can result from specific sources of external exposure associated with residence, occupation, or lifestyle, or from all persons having similar, more or less random environmental exposures, but differing in uptake and excretion. Fei et al. (2007) report that higher exposure was associated with young maternal age, low parity, elevated BMI, and being a nonsmoker. Age, parity, and BMI may well reflect interindividual differences in uptake and excretion rather than differences in exogenous environmental exposure. We have much to learn about the pathways leading to elevated blood levels of PFOA and PFOS, but to the extent that metabolic differences among individuals determine their blood levels, such differences could also produce concomitant variation in birth weight and infant adiposity. Hypothetically, if maternal physiology influenced both exposure biomarkers and blood glucose levels, for example, biomarkers in exposure and outcome would be associated due to a common influence, rather than exposure affecting outcome. Although this is highly speculative, tendencies in this direction could well account for the modest associations between exposure biomarkers and biological variability in infant size.

The challenge faced by these and future investigators is to isolate causal effects of the exposure on the outcome from shared intrinsic determinants producing noncausal associations, that is, confounders. To the degree that these shared influences can be measured, they can be controlled statistically. The magnitude of change from simply controlling for the few markers of exposure that are independently related to health outcomes markedly attenuated the risk estimates in both of the studies, nonetheless leaving discernible indications of a weaker association. Several strategies could help future studies to distinguish shared biological determinants of measured exposure and outcome from a causal effect: First, examine an exposure distribution that extends above the range of normal variation so that blood levels reflect interindividual differences in exogenous exposure and not just individual differences in uptake and excretion. Second, conduct studies that are large enough to address clinically significant outcomes, including those that fall outside the range of normal biological variability. Finally, identify and adjust for improved markers of maternal and fetal physiology that may influence measured blood levels of the exposure and independently affect the health outcomes of interest.

## Figures and Tables

**Figure:**
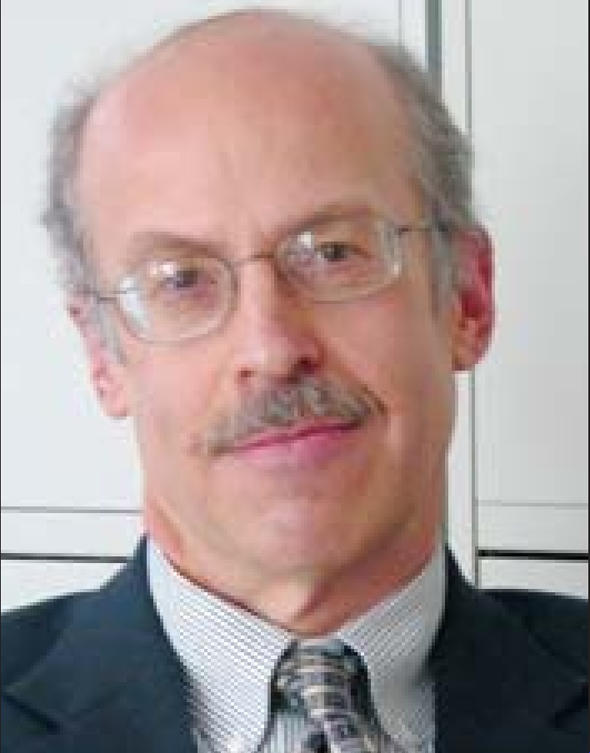
David A. Savitz
